# Evaluating factors impacting early career physician-scientists’ decisions to continue research careers in the United States of America

**DOI:** 10.1186/s12909-025-07144-4

**Published:** 2025-04-17

**Authors:** Kassem Farhat, Aleksandar Obradovic, Aisha Siebert, Han Naung Tun, Evan K. Noch, Jennifer M. Kwan

**Affiliations:** 1 Department of Internal Medicine, Yale School of Medicine, New Haven, CT USA; 2 Department of Medicine, Columbia University, New York, NY USA; 3 Boston Children’s Hospital, Harvard Medical School, Boston, MA USA; 4 Larner College of Medicine, University of Vermont, Burlington, VT USA; 5 Department of Neurology, University of Texas Southwestern Medical Center, 6000 Harry Hines Blvd, Dallas, TX 75235 USA; 6 O’Donnell Brain Institute, UT Southwestern Medical Center, Dallas, TX USA; 7 Section of Cardiovascular Medicine, Yale School of Medicine, 300 George St Ste 759, New Haven, CT 06511 USA

**Keywords:** Physician-scientist, Research, Early-career, Medical education, Training, Workforce

## Abstract

**Background:**

Bridging the gap between laboratory discoveries and patient care relies heavily on the physician-scientist workforce, which has historically served as a cornerstone in advancing biomedical sciences. However, the past decade has witnessed a remarkable decline in the number of emerging physician-scientists, raising concerns about the future of this vital community. This study aimed to evaluate the current state of early career physician-scientists on a national scale and explore challenges that hinder its growth, thereby limiting potential scientific innovation and progress.

**Methods:**

A survey was conducted in the United States and distributed to 110 nationally representative institutions using an online platform (SurveyMonkey), targeting physician-scientists at their late stage of clinical training (residents/fellows) and graduates of training programs within the past 10 years. 265 submitted results but after filtering for incomplete responses, a total of 230 survey results were used in the analysis. The survey evaluated scientific career trajectories, challenges encountered, and top priorities. Statistical analyses, including Chi-square and Fisher’s exact tests, were used to compare differences between groups.

**Results:**

A total of 230 physician-scientists completed the survey. The respondents were predominantly assistant professors (46%), while 27% were still enrolled in career training programs. Nearly half of the participants reported considering leaving their research career within the next two years. The primary reasons cited for this included burnout and unhappiness (35%), stress (35%), and lack of funding (30%). The most frequently reported career challenges were achieving a balance between clinical and educational responsibilities (63%) and maintaining work-life balance (53%), followed by insufficient research funding (41%). Additionally, participants underscored key factors they prioritize when seeking employment, including hybrid research-clinical opportunities (67%), work-life balance (52%), and financial security (26%).

**Conclusion:**

This national survey provides an overview of the current state of early-career physician-scientists. It examines the factors contributing to the inclination to leave the scientific track and identifies the primary career challenges faced by this vulnerable community. Furthermore, it highlights key priorities of physician-scientists and gaps that require attention, offering valuable insights into strategies for retaining and supporting this critical workforce.

**Supplementary Information:**

The online version contains supplementary material available at 10.1186/s12909-025-07144-4.

## Background

Physician-scientists are highly trained clinicians who combine their medical expertise with rigorous research training, translating their scientific discoveries into clinical practice by dedicating a substantial portion of their time in laboratories [1]. 

Over the years, advocacy initiatives and task forces have sought to address the growing challenges hindering the physician-scientist workforce [2]. These efforts have provided key recommendations to policymakers to tackle critical issues threatening this vital community [3]. Building policies that protect the career trajectories of physician-scientists while fostering the recruitment of bright minds to embark on this journey is of high importance, particularly in today’s rapidly evolving healthcare landscape with its associated complexities [4]. Ensuring a robust pipeline for developing visionary, independent physician-scientists requires not only improved recruitment strategies but also effective retention efforts [5]. To achieve this goal, addressing pertinent obstacles, including inadequate compensation, increasing clinical demands, and decreasing research funding, is imperative [5, 6]. 

Historically, advocacy efforts have successfully driven cultural shifts, as evidenced by the increased U.S. National Institutes of Health (NIH) budget and private sector initiatives to preserve the physician-scientist workforce [1, 7]. However, in recent years, stagnation in funding growth and failure to keep pace with the rising research costs have compounded the ongoing challenges faced by this community [8]. Additionally, difficulties balancing work-life responsibilities, when added to the equation, further exacerbate these issues, creating additional barriers to career sustainability [9]. A recent report by Garrison et al. analyzing changes in the physician-scientist workforce between 2011 and 2020 has shed light on these challenges [2]. The report revealed alarming trends despite a growth in MD-PhD program enrollment. This included a significant decline of interest in pursuing a research career among graduating medical students and a growing number of individuals leaving research as their primary professional activity [2]. The physician-scientist workforce working group report released by the NIH in 2014 underscored another troubling data that shows an aging community with a substantial decline in physician-scientists between 31 and 50 years [5]. These trends raise serious concerns about the long-term viability of the community. Of particular concern is the transition from clinical training to junior faculty positions, which has been identified as the leakiest point in the pipeline [8]. Early-career physician-scientists face unique challenges, making them especially sensitive to systemic changes, thereby warranting focused intervention [8]. 

In this survey, we aim to deliver an update on the current state of physician-scientists, with a focus on early career investigators, on a national scale. In addition, we strive to explore their perspectives on key factors affecting the future of this workforce, contributing to the development of a strategic roadmap to preserve and strengthen this community.

## Methods

The study survey was designed to incorporate demographic data (Age, Level of Training, Gender, Sexual Orientation, Race, Medical Specialty, Geographic Region, and Year of Graduation from Terminal Level of Training), as well as response to questions regarding career development support, breakdown of clinical and research responsibilities, funding, and perceived career challenges, including the likelihood of leaving academic medicine and reasons for doing so. This survey was disseminated by email to department chairs at 110 institutions across the United States to be distributed to their early career investigators. The survey tool developed can be found in the supplementary material (Supplementary Material S1).

After filtering incomplete responses for which demographic data were missing, 230 responses were compiled into an integrated data table. We initially assessed the pairwise associations between all demographic and binarized response variables using Fisher’s Exact test, with *p*-values corrected using the Benjamini-Hochberg method. By this approach, a strong correlation was found between variables with strong semantic association, as expected (e.g. age correlated with years since graduation from terminal degree and correlated with level of training; lack of funding as a perceived career challenge correlated with citing concern over lack of funding as a reason to leave academic medicine). However, geographic region was found to be strongly associated with citing under-compensation as a reason to consider leaving their current position, with 19/38 (50%) of respondents localized in the Southwest (defined as Arizona, California, Colorado, Nevada, New Mexico, and Utah) reporting concern over under-compensation, as compared to 31/190 (16%) of respondents from other regions. We then performed a focused analysis on response variables regarding perceived career challenges and reasons given for considering leaving academic medicine, stratified by Gender and by Under-Represented Minority status (As defined by NIH criteria). By Fisher’s Exact Test, with Benjamini-Hochberg correction of *p*-values, there were no significant differences in any response variable between men and women, and only one response variable with a statistically significant difference when stratified by Under-Represented Minority Status. Statistical analyses were performed in R v4.2.3 programming language in RStudio v2024.04.1 integrated development environment.

## Results

The survey was distributed to institutional leaders for dissemination, resulting in 334 responses in total, including incomplete submissions, with 230 completed surveys, yielding a completion rate of 69%. Among respondents, 56% were male, with the largest represented age group being 35 to 44 years (67%). The study included physician-scientists at various career stages, with assistant professors comprising the largest group (40%), followed by fellows (27%), residents (14%), associate professors (7.5%), instructors (7.5%), and full professors (1.9%) (Table 1).

**Table 1:** Sociodemographic characteristics of respondents Physician-Scientists

Characteristics		Respondents *N* (%)
Age (years)	25–34	45 (20%)
	35–44	154 (67%)
	45–54	29 (13%)
	55–64	1 (0.5%)
	65+	1 (0.5%)
Level of training	Assistant Professor	64 (40%)
	Associate professor	12 (7.5%)
	Professor	3 (1.9%)
	Instructor	12 (7.5%)
	Other	4 (2.5%)
	Fellow	43 (27%)
	Resident	22 (14%)
Gender	Female	97 (43%)
	Male	128 (56%)
	Prefer to self-describe	2 (0.9%)
Hispanic	No	206 (92%)
	Yes	16 (7.1%)
	Other	3 (1.3%)
Sexual orientation	Bisexual	3 (1.3%)
	Gay male	13 (5.8%)
	Prefer to self-describe	5 (2.2%)
	Queer	1 (0.4%)
	Straight/heterosexual	201 (90%)
Race	Asian	57 (26.1%)
	Black or African American	4 (1.8%)
	Multi-racial	11 (5%)
	White	135 (61%)
	Other	15 (6.8%)

**Fig. 1:**
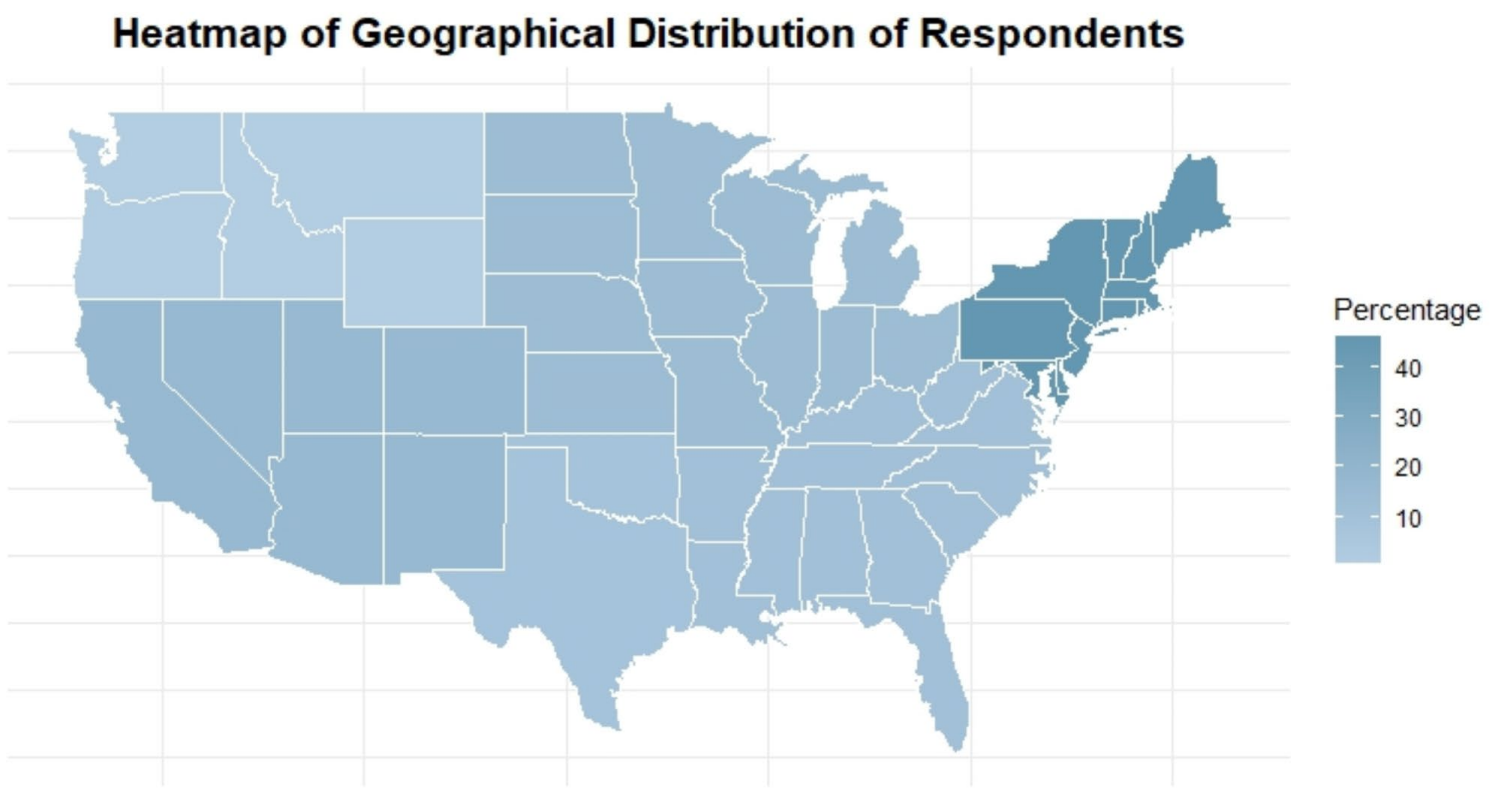
Heatmap of geographical distribution of respondents

Respondents were categorized into four broad medical fields, including primary care/medicine-based (65%), surgical (6%), diagnostic (27%), and acute care subspecialties (2%) (Table 2). The detailed distribution of physician-scientists across these specialties is shown in the supplementary Table 1. In addition, racial demographics revealed that the majority identified as White (61%), followed by Asian (26.1%), and African American (1.8%). Hispanics accounted for only 7.1% of the sample. Regarding sexual orientation, 90% identified as straight/heterosexual, 5.8% as gay male, 1.3% as bisexual, and 0.4% as queer. Geographically, the Northeast was the most represented region (46%), followed by the Southwest (17%), the Midwest (14%), the Southeast (11%), the South (9.2%) and the Northwest (0.9%). A heatmap illustrating geographic distribution is shown in Fig. 1, and additional demographic characteristics are detailed in Table 1 and supplementary Table 2.

**Table 2:** Representation of respondents across different specialty groupings

Specialty groupings	Respondents *N* = 230 (%)
Primary Care/Medicine-Based Subspecialties	148 (65%)
Surgical Subspecialties	17 (6%)
Diagnostics Subspecialties	61 (27%)
Acute Care Subspecialties	4 (2%)

This table summarizes the distribution of respondents across various specialty groupings. A detailed breakdown of specialties within each subgroup is provided in supplemental Table 1

Among respondents, 185 out of 230 reported their current research and clinical work ratio. The most common ratio was 80/20, reported by 37% of participants, followed by 75/25 (18%). A smaller proportion maintained an even balance of 50/50 (9%). Notably, 13% of respondents were in full-time clinical roles, while 6% dedicated 100% of their time to research. The remaining respondents had mixed allocations, with 8% at 60/40, 6% at 25/75, and 3% at 40/60.

Regarding respondents’ preferred research-clinical balance, perspectives varied significantly. Those dedicating more than 75% of their time to research expressed a strong preference for research, citing the need for full-time dedication to manage lab work, grant applications, and research demands. Among those who continued clinical practice, 20–25% clinical time was generally considered sufficient to maintain meaningful patient engagement while focusing on their research careers.

In contrast, among those with less than 75% research time, three distinct groups emerged: (1) the majority, who preferred more than 75% research time but were constrained by clinical demands or financial security and represent the majority (2), those who sought an even balance to preserve their clinical skills and patient care while maintaining research productivity, and (3) a minority who had fully transitioned to clinical work early in their careers, citing reasons such as financial stability, career constraints, or the burden of research funding efforts.

Following graduation, the most sought-after career paths were hybrid clinical research positions (43%), followed by academic research positions (17%), clinical roles (8.6%), further postgraduate training (3.2%), and industry positions (0.5%). (Table 3) Notably, 25% of respondents were still in training at the time of the survey (Table 3).

**Table 3:** Career focus and achievements of survey respondents

Career Characteristics		Respondents *N* = 230 (%)
Receipt of Foundation Award During Junior Faculty Appointment		
	Yes	88 (43%)
	No	142 (57%)
First Position Following Completion of Clinical Training		
	Academic Center (Clinical Position)	19 (8%)
	Academic Center (Hybrid Research/Clinical Position)	96 (42%)
	Academic Center (research position)	37 (16%)
	An Additional Postgraduate Residency/Fellowship	7 (3%)
	Still in Training (Residency/Fellowship)	58 (25%)
	Industry (Pharma, Med tech, Digital tech, etc.)	1 (1%)
	Private practice	2 (1%)
	Other	10 (4%)
Primary Area of Intended Professional Career Focus		
	Administration	5 (2%)
	Basic Research	71 (31%)
	Clinical Duties	21 (9%)
	Clinical Research	34 (15%)
	Education	5 (22%)
	Therapeutics/Diagnostic Development	5 (2%)
	Translational Research	86 (37%)
	Other	3 (1%)

Furthermore, 90% reported receiving career development award (CDA) application support from their department leadership. However, despite this support, fewer institutions offered salary equalization between clinicians and physician-scientists. A similar pattern was observed for research incentives and RVU adjustments, where institutional support remained comparatively limited. Further details are provided in Table 4.

**Table 4:** Institutional support for career development awards, salary equity, and research incentives

	Number (%)
Does your current department/chair support your application to career development awards (like a NIH K award, DOD, foundation, specialty society)?	
NO	13 (6%)
YES	205 (90%)
Unsure	10 (4%)
Does your current department/chair equalize base salaries between full-time clinicians and physician-scientists?	
NO	53 (36%)
YES	53 (36%)
Unsure	43 (28%)
Does your current department/chair provide research incentives or research RVUs?	
NO	86 (59%)
YES	35 (24%)
Unsure	25 (17%)

Additionally, only 43% of participants reported receiving funding or CDAs during their junior faculty years. Regarding the number of attempts to secure a successful award, a total of 154 responses were recorded: 32% had not yet applied, 38% secured funding on their first attempt, and 21% required two attempts. A minority required multiple attempts, with 5% obtaining funding after three attempts and 3% after more than four attempts.

Notably, among 119 respondents, 52% (62/119) reported restrictions on research time requirements imposed by foundation or specialty society awards (> 70% commitment), while 31% indicated no such restriction, and 17% had not yet applied.

Irrespective of initial professional trajectory, the majority of respondents intended to focus their professional efforts on translational (38%) and basic (31%) research, and smaller proportions prioritized clinical research (14%) and patient care (9.2%). Additional details are presented in Table 3.

**Fig. 2:**
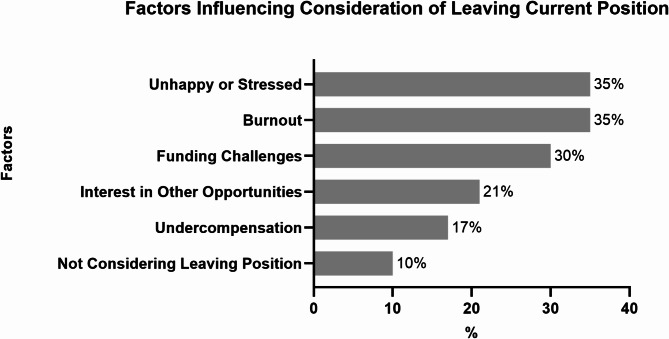
Key reasons to consider leaving current position

**Fig. 3:**
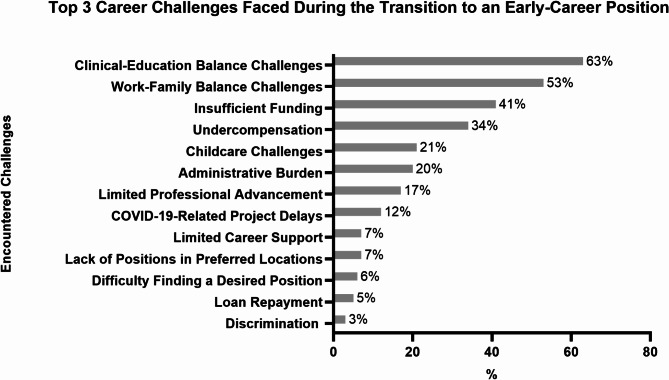
Challenges encountered during the transition to an early-career faculty position

This study reveals that nearly half of respondents (49%) had considered leaving academic medicine within the next two years over the past six months, despite 65% expressing a high likelihood (> 75%) of remaining in the field over the next five years. Among those considering departure, the majority (68%) were from primary care and medicine-based specialties (Table 5). The most represented groups included hematologists/oncologists (13%), pulmonologists and critical care specialists (12%), and infectious disease physicians (11%) (Supplementary Table 4). Additional details on the distribution of respondents across different specialties are available in Supplementary Table 1. The primary reasons cited for considering departure included burnout or unhappiness (35%) and stress (35%), followed closely by funding challenges (30%) (Fig. 2). When examining broader career challenges, respondents most frequently reported difficulties balancing clinical and scientific responsibilities (63%), work-family balance (53%), and limited funding opportunities (41%). Further challenges are outlined in Fig. 3. In this context, as a counterforce, 63% (145/230) of respondents expressed interest in joining advocacy organizations, while 24% were unsure, and 13% were not interested.

**Table 5:** Specialty distribution of respondents considering leaving research careers

Specialty groupings	Respondents *N* = 114 (%)
Primary Care/Medicine-Based Subspecialties	78 (68%)
Surgical Subspecialties	6 (5%)
Diagnostics	30 (26%)
Acute Care Subspecialties	1 (1%)

This table summarizes the distribution of respondents considering leaving research careers across various specialty subgroupings. A detailed breakdown of specialties within each subgroup is provided in supplemental Table 4

Notably, no significant gender differences were observed regarding work-life balance concerns (*p* = 0.15). However, non-minority groups more frequently identified funding as a barrier compared to underrepresented groups (*p* = 0.045). Regional differences were also evident, with concerns about compensation being particularly pronounced in the Southwest. Half of respondents from this region cited under-compensation as their primary reason for considering leaving their current position, a significantly higher proportion compared to other regions (50% vs. 16%; *p* = 0.000022).

**Fig. 4:**
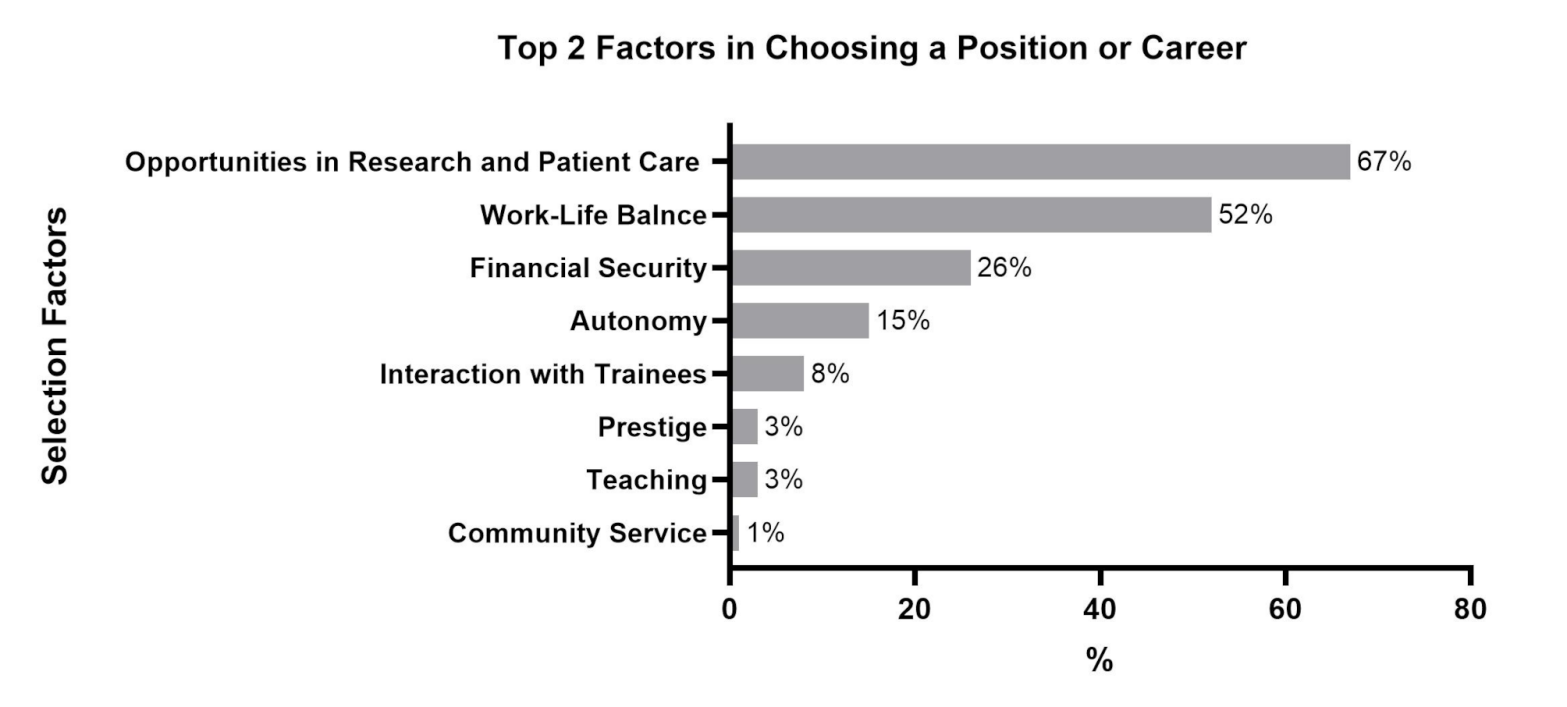
Important factors when choosing a position or career

The majority of respondents (67%) identified opportunities for research and patient care as the most critical job selection criteria, followed by work-life balance (52%), financial security (26%), autonomy (15%), and interaction with trainees (8%) (Fig. 4).

## Discussion

Nearly four decades separated two prominent former directors of the National Institutes of Health (NIH) in voicing their concerns about the future of the scientific workforce. In 1979, Dr. James Wyngaarden famously described physician-scientists as an “endangered species” during a symposium addressed to the Committee on Medical Education of the New York Academy of Medicine [2]. Decades later, in 2014, Dr. Francis Collins revisited the issue, analyzing the composition and size of the physician-scientist community and expressing equally profound concerns about its state and long-term sustainability [10]. 

The current survey offers a comprehensive overview of the demographics, career trajectories, and challenges of early-career physician-scientists on a national scale. The findings presented here highlight endangers encountered by this workforce, which could jeopardize its sustainability and impede scientific progress. Historically, the physician-scientist community has confronted numerous obstacles, including a paucity of funding opportunities and a lack of robust policies and incentives aimed at workforce retention [2, 6]. To mitigate the devastating long-term consequences of career challenges, which, if not appropriately addressed, are likely to destabilize the workforce over the next decade, it is crucial to implement comprehensive measures that establish a robust infrastructure [11]. Such measures include, but are not limited to, expanding early-career grant programs and institutionally supported bridge funding, providing financial incentives, ensuring protected research time, and developing structured mentorship and career development programs. These initiatives would support skilled young investigators, who often compete with well-established scientists in harsher work environments and limited career-launching opportunities.

A diverse workforce is essential for advancing scientific innovation, as it fosters a broader range of inquiry, produces highly impactful research, and enhances the inclusion of marginalized communities in clinical trials [12, 13]. However, significant disparities remain within the physician-scientist community, affecting underrepresented racial and ethnic minorities, sexual and gender minorities, individuals of low socioeconomic status, and individuals with disabilities [12–16]. For instance, in a previous study that included 44,433 predoctoral physician-scientists, we demonstrated a White predominance (67%), alongside an underrepresentation of Hispanic (6%) and African American (4.1%) individuals [14]. This underrepresentation is subsequently mirrored by a decline in the participation of Hispanic and African American physicians in clinical academic medicine following graduation [17]. Similarly, the 2014 NIH Physician-Scientist Workforce Group report highlighted the underrepresentation of African American, Native American, and Hispanic individuals in medicine, comprising only 7% of the total applicant pool and 4.7% of awardees for NIH research project grants [10]. 

Findings of the current study echo these reports, revealing a similar distribution among early career physician-scientists, with 26.1% identifying as Asian, 7.1% as Hispanic, and only 1.8% as African American. This persistent underrepresentation is rooted in systemic barriers encountered early in the training pipeline, including financial limitations, lack of mentorship, and limited research interest in basic and translational work, particularly among African American and Hispanic individuals [13, 14, 18, 19]. In fact, coming from economically leveraged backgrounds, coupled with the under-compensation of physician-scientists compared to their clinical peers, underrepresented individuals in medicine (URM) are actively disheartened from pursuing and devoting their time to research [20]. 

To address these disparities, it is crucial to develop robust peer networking and longitudinal mentorship programs tailored to connect URM trainees with mentors from diverse backgrounds whom they can learn from, look up to, and gain different perspectives to support them in navigating their research careers [21, 22]. Additionally, it is imperative to expand NIH funding initiatives, such as loan repayment programs, to alleviate financial burdens and encourage commitment to research careers [12, 23]. 

Furthermore, despite a cultural shift in the United States that has made the LGBTQ + community more comfortable with openly expressing their gender identity and sexual orientation, this group remains underrepresented in medicine and faces significant stigma, discrimination, and bias despite their significant contributions in different scientific fields [23–26]. Our previous work examining the status of LGBTQ + trainees highlighted sexual harassment as a major barrier to career advancement and underscored their underrepresentation in the community, aligning with data from the current study [24]. Our findings also highlight significant demographic disparities, with the Northeast being overrepresented (46%) compared to other geographic regions. This trend parallels broader geographic disparities in the distribution of physician-scientists, which may be partly explained by the East Coast’s long-standing history as a hub for prestigious, well-established academic institutions and strong research infrastructures that attract top talent and provide abundant career opportunities for physician-scientists [27]. 

Over half of this study’s respondents graduated in 2020 or later, with the majority (81%) comprising assistant professors and trainees (residents and fellows). This distribution reflects the study’s focus on early-career physician-scientists.

Career attrition is evident at multiple stages of the pipeline, particularly during training and early career phases, which are identified as the primary leakage points [28, 29]. Although our survey did not include students, it is estimated that up to 10–15% of MD-PhD students drop out before completing their degrees [28]. For those who transition away from research, many redirect their focus to patient care in academic settings, while others move to industry, private practice, or accept tenure-track positions but allocate minimal time to research [10]. Additionally, nearly half of our study respondents (49%) reported considering leaving academic medicine within the next two years. While troubling, this finding is neither surprising nor limited to early career investigators as it aligns with broader trends observed at every pipeline stage [29]. These concerns are particularly relevant given the significant decline in the physician-scientist community over the past three decades, with the prevalence of physicians devoted to research dropping from 4.5% in 1985 to 1.6% in 2011, despite the doubling of the NIH budget [15, 30]. However, despite contemplating leaving, the majority of our respondents also reported a strong likelihood of continuing their research careers. This may be driven by their strong commitment to scientific discovery and the meaningful impact of their work. Additionally, secured funding, even amid ongoing grant-writing challenges, may provide temporary stability. Others may remain hopeful for improvements in the research environment, while some may not yet feel ready to transition into more clinical roles or industry positions.

This outflow, as outlined by the NIH Physician-Scientist Workforce Working Group report, has been attributed to increased dropout rates among dual-degree (MD-PhD) physician-scientists and decreased enrollment of MD-only physician-scientists [10, 31]. Consequently, these shifts have further led to the aging of the physician-scientist community. Hence, the number of investigators in their 40s has plummeted from approximately 7,000 to 3,800, while the proportion of older investigators has steadily increased [31]. 

Moreover, CDAs are crucial for young investigators, offering essential funding to support independent research projects and aiding their transition to independent careers [6]. However, less than half (43%) of our respondents reported receiving such funding during their junior faculty years. This is worrisome, as the lack of support at this critical stage may jeopardize the ability of this generation to successfully launch and sustain their research careers, thereby leading to a huge national loss in intellectual capital [32]. Even among those who receive CDA, up to one-third fail to progress and obtain their first independent NIH R01 grant [29]. These challenges are translated by the increased average age at which physician-scientists receive their first NIH R01 grant by 10 years between 1980 and 2011, which eventually will stretch their early-career phase [10]. 

Among respondents contemplating a career shift, burnout or unhappiness (35%) and stress (35%) were identified as the leading reasons. This is likely driven by the pressure of navigating multiple roles, particularly when job demands exceed available resources in both clinical and research settings [33]. Additionally, unforeseen external factors can significantly exacerbate these conditions. For example, our group previously investigated the impact of the COVID-19 pandemic, a force majeure that disrupted nearly all aspects of life, uncovering heightened stress levels, decreased productivity, and reduced optimism within the physician-scientist community [34, 35]. To address these issues, organizations must adopt strategies that enhance resilience and foster a culture of well-being by providing resources and support tailored to the needs of physician-scientists.

On the other hand, financial constraints, such as funding scarcity and under-compensation, surfaced as another significant reason behind a career change among our respondents (30%). Financial instability remains a major barrier to career sustainability and a key driver of attrition across all stages [6, 8]. To highlight this issue, our data indicate a decline in institutional financial commitments beyond CDA application assistance, reflected in the limited equalization of base salaries with clinical fellows and minimal research incentives. Notably, respondents from the Southwest were disproportionately more likely to consider leaving academia, citing under-compensation as a primary factor contributing to their dissatisfaction. This trend likely results from a mismatch between compensation and the region’s cost of living.

Moreover, protected research time is essential for sustaining research careers. Most CDAs, including K01, K08, K22, K23, K25, Parent K99, and MOSAIC K99, require a minimum commitment of nine-person months (75% of full-time professional effort). However, 52% of respondents (62/119) reported restrictions on the required research time, posing a significant barrier to pursuing a dedicated research career. This highlights the urgent need for institutional policy changes that guarantee a minimum percentage of protected research time, supplemented by appropriate salary support.

Addressing challenges at all stages of the pipeline, particularly at the entry-level where individuals are most vulnerable, could mitigate the high attrition rates and ensure the long-term sustainability of the physician-scientist workforce. The top cited among our respondents was balancing clinical and scientific responsibilities (63%). This struggle often stems from the difficulty of allocating sufficient time to both research and direct patient care, as many physician-scientists value the blend of these experiences and find them highly rewarding [9, 33]. Yet, despite the lack of financial incentives or promotion opportunities, many physician-scientists express pride and a sense of privilege in training and mentoring the next generation [9, 33]. Guiding trainees as they embark on this challenging yet fulfilling career path is seen as an integral part of their role, underscoring the dedication and commitment of this workforce [9, 33]. 

Furthermore, the lengthy training period, the heightened pressure to maintain research productivity for career advancement, and financial challenges linked to repaying debt and career under-compensation are among the many factors that strain the work-family balance for physician-scientists [33]. This challenge was reported by more than half (53%) of our respondents. As a result, individuals contemplating starting a family may either give up on that dream or delay this important step. This decision can be particularly complex for women physician-scientists, for whom timing may be more critical due to health risks related to delaying childbearing [9, 36]. Consequently, some may have chosen to prioritize either research or clinical work to dedicate more time to family responsibilities. Successfully managing these competing demands requires not only exceptional time-management skills but also a supportive environment [37]. This includes support at the family level and institutional policies that provide essential services, such as childcare assistance, flexible schedules, and maternity support [9, 37]. 

Ultimately, all the aforementioned translate into a wide workforce desire for career stability by seeking job opportunities that provide access to research and clinical work while ideally preserving work-life balance and ensuring financial security. As explicitly reported by our respondents, these key elements have the potential to significantly reduce burnout and alleviate stress [38]. 

Amid recent policy changes, particularly those affecting research funding, which threaten the longevity of the research community and discourage young investigators from pursuing this path, advocacy campaigns and non-profit organizations such as the American Junior Investigator Association (AJIA) will play a crucial role in empowering this community and serving as a primary voice against emerging challenges. In this context, while 63% of our respondents expressed interest in joining such organizations, this number is expected to rise in the near future. Lastly, given the quantitative nature of survey data, a mixed-methods approach integrating surveys with focus groups and interviews would offer greater breadth and depth in understanding the challenges faced by physician-scientists with more insight into personal experiences.

## Limitations

This study has several limitations. First, its cross-sectional design limits the ability to assess temporal changes, and the absence of longitudinal follow-up prevents the evaluation of how participants’ experiences and career trajectories evolve over time. Future research incorporating longitudinal methodologies would provide a more comprehensive understanding of trends, the persistence of challenges, and the long-term impact on early-career physician-scientists. Another limitation is that the relatively small number of respondents (*n* = 230) may limit the generalizability of the findings. Within this context, our study’s regional distribution of respondents suggests a potential overrepresentation of the Northeast and an underrepresentation of the Northwest, likely influenced by institutional dissemination methods and voluntary participation. Regional classification may have also contributed to disparities. However, we do not anticipate a significant impact on the overall conclusions. The Northeast’s higher representation aligns with its dense concentration of research institutions, and we believe that the percentage of physician-scientists considering leaving their research careers in the Southwest will remain consistent with broader trends. Future studies incorporating direct recruitment strategies or targeted outreach to underrepresented regions may help enhance geographic balance and improve the generalizability of findings. Third, selection bias may have influenced our findings, as individuals with stronger opinions about physician-scientist career challenges may have been more likely to respond. Lastly, this study lacks formal validation for the survey. Although it was informed by relevant literature and expert input, no pre-testing or reliability assessment was conducted, which may impact the reproducibility and generalizability of the findings. Despite this limitation, the study offers valuable insights into the current landscape and identifies key challenges that warrant further investigation in larger, more diverse cohorts.

## Conclusion

This survey provides valuable insights into the current state of early-career physician-scientists, a vital community for scientific advancement. It highlights challenges that threaten the future of this workforce, emphasizing the urgent need for a strategic roadmap that integrates institutional, governmental, and organizational efforts to preserve and strengthen this critical community.

## Electronic supplementary material

Below is the link to the electronic supplementary material.

Supplementary Material 1

Supplementary Material 2

Supplementary Material 3

Supplementary Material 4

Supplementary Material 5

## Data Availability

The datasets used and/or analyzed during the current study are available from the corresponding author on reasonable request.
