# Supplementary material for: Evaluating factors impacting early career physician-scientists’ decisions to continue research careers in the United States of America
Source: BMC Med Educ. 2025 Apr 17;25:564. doi: 10.1186/s12909-025-07144-4 (PMC12007356; doi:10.1186/s12909-025-07144-4)
Supplement: Supplementary file 1 — Supplementary Material 1 [file 12909_2025_7144_MOESM1_ESM.pdf]

Outlook and Support of Early Career Physician-Scientists

Consent

* 1. Physician-scientists represent only 1.5% of the biomedical workforce (Jain et al 2019) and face unique challenges. Transitioning from the resident and fellow to junior faculty career stages are the leakiest parts of the physician-scientist pipeline.

This is a survey on behalf of the American Junior Investigators Association (AJIA), a nonprofit dedicated to the success of early career physician-scientists across the full spectrum of specialties. This survey is intended to be distributed to late stage PSTP/research track residency/fellowship trainees/graduates of MD PhD and DO PhD programs and those who have graduated from PSTPs/research track residencies/fellowships within the past 10 years in the United States.

The goal of the survey is to assess how our recent research track trainees are faring and what drives their decisions in choosing faculty positions. The COVID-19 pandemic has further compounded the above issues, and we want to prepare for and mitigate potential problems that may require advocacy/education/outreach for clinical and research directions to avoid losing a generation of physician-scientists.

Through the survey, we seek to characterize how early-career physician scientists have fared, important factors in choosing a career and any challenges/problems they are facing in their transition to a full time position post training. The results of the survey will be used to advocate for changes to policies/institutional structures that will help retain and advance a diverse academic medical faculty. Any significant results will potentially be published in a peer-reviewed journal.

This survey is completely anonymous and voluntary. No personally identifying information will be linked to your survey responses. There is no harm or risk associated with taking the survey.

To protect the survey taker, the account in which responses are stored is password-protected and can only be accessed by the researchers.

The survey will take approximately 5-7 minutes to complete.

If you have any questions, please feel free to contact the following research coordinators of this study: Yale School of Medicine research coordinator of this study: Jennifer M Kwan, MD PhD jennifer.kwan@yale.edu, the University of Texas, Southwestern Medical Center research coordinator, Evan Noch MD PhD, evan.noch@utsouthwestern.edu or the Massachusetts General Hospital research coordinator, Dania Daye MD PhD. The Survey has been IRB approved at Massachusetts General Hospital.

*Agreement of Subject I have read the above information and understand the terms of my participation. I agree to participate in this study.

◯ Yes

◯ No

Outlook and Support of Early Career Physician-Scientists

2. What is your age (in years)

- 25-34
- 35-44
- 45-54
- 55-64
- 65+
- Other (please specify)

3. What is your level of training

- Resident
- Fellow
- Instructor
- Assistant Professor
- Associate professor
- Professor
- Other (please specify)

4. Gender identity

- Female
- Male
- Transfemale
- Transmale
- Queer
- Genderqueer
- Prefer to self describe

5. Sexual orientation

- Straight/heterosexual
- Gay male
- Lesbian
- Bisexual
- Queer
- Prefer to self describe

6. Are you Hispanic, Latino or Spanish origin?

- Yes
- No
- Other (please specify)

7. Are you:

- White
- Black or African American
- American Indian or Alaska Native
- Asian Indian
- Chinese
- Filipino
- Japanese
- Korean
- Pakistani
- Vietnamese
- Native Hawaiian
- Guamanian
- Chamorro
- Samoan
- Multi-racial
- Other (please specify)

8. What is your specialty?

9. Region of country

10. What was your year of graduation from your terminal training (ie clinical fellowship) before searching for a full-time position?

- prior to 2015
- 2015
- 2016
- 2017
- 2018
- 2019
- 2020
- 2021
- 2022
- 2023
- 2024
- N/A
- Other (please specify)

11. How many years has it been since you graduated from your MD PhD/DO PhD or MD/DO programs?

- ○ 0
- ○ 1-3
- ○ 3-6
- ○ 7-8
- ○ 9-10
- ○ 11+
- ○ N/A
- ○ Other (please specify)

12. How many years has it been since you graduated from your PhD program if applicable?

- ○ 0
- ○ 1-3
- ○ 3-6
- ○ 7-8
- ○ 9-10
- ○ 11+
- ○ N/A
- ○ Other (please specify)

13. What is your PREFERRED research to clinical ratio (research % / clinical %)?

- ○ 100/0
- ○ 80/20
- ○ 75/25
- ○ 60/40
- ○ 50/50
- ○ 40/60
- ○ 25/75
- ○ 0/100
- ○ N/A
- ○ Other:

14. Why is this your PREFERRED research to clinical ratio?

15. What is your CURRENT research to clinical ratio (research % / clinical %)?

- ◯ 100/0
- ◯ 80/20
- ◯ 75/25
- ◯ 60/40
- ◯ 50/50
- ◯ 40/60
- ◯ 25/75
- ◯ 0/100
- ◯ N/A
- ◯ Other:

16. Does your current department/chair support your application to career development awards (like a NIH K award, DOD, foundation, specialty society)?

- ◯ Yes
- ◯ No
- ◯ Other:

17. Does your current department/chair equalize base salaries between full time clinicians and physician-scientists?

- ◯ Yes
- ◯ No
- ◯ Other:

18. Does your current department/chair provide research incentives or research RVUs?

- ◯ Yes
- ◯ No
- ◯ If yes, please provide examples of how this is awarded

19. Which NIH or VA career development award (CDA) have you applied for and/or received?

- [ ] K08 APPLIED
- [ ] K08 RECEIVED
- [ ] K99/R00 APPLIED
- [ ] K99/R00 RECEIVED
- [ ] K23 APPLIED
- [ ] K23 RECEIVED
- [ ] other K awards (K12, KL2, etc)
- [ ] Other (please specify)
- [ ] VA CDA: APPLIED
- [ ] VA CDA: RECEIVED
- [ ] DOD: APPLIED
- [ ] DOD: RECEIVED
- [ ] I have received a R01 or equivalent
- [ ] N/A

20. Were you funded by a foundation award/career development award (CDA) in your junior faculty position?

- ◯ Yes
- ◯ No

21. If you have a career development award, how many times did you have to apply for your current successful award?

- ◯ 1
- ◯ 2
- ◯ 3
- ◯ ≥4
- ◯ Other (please specify)

22. If you have a non NIH/VA CDA, what foundation or specialty society is your CDA from?

- ◯ Doris Duke
- ◯ Burroughs Wellcome Fund
- ◯ American heart association
- ◯ AACR
- ◯ Other (please specify)
- ◯ ASCO
- ◯ IDSA
- ◯ ATS
- ◯ RSNA (Radiologic Society of North America)

23. What is the name of the above mentioned award?

24. What is the length of the foundation/specialty society award in years?

- 1
- 2
- 3
- 4
- 5
- Other (please specify)

25. What is the award amount total?

26. Is the award mainly for salary?

- Yes
- No
- Other (please specify)

27. Is there protected research time restrictions for your foundation/specialty society award (>70% research requirement)?

- Yes
- No
- Other (please specify)

* 28. After you completed residency/fellowship, what was your first position?

- Academic center (research position)
- Academic center (clinical position)
- Academic center (hybrid research/clinical position)
- Industry (pharma, med tech, digital tech, etc)
- Private practice
- An additional postgraduate residency or fellowship
- I'm still in my residency/fellowship
- Other (please specify)

* 29. What are the top 3 challenges you have encountered thus far in your early faculty career/transition to early-career faculty?

- [ ] Lack of opportunity/funding
- [ ] Not finding a position in desired location
- [ ] Not able to find a desired position
- [ ] Loan repayment
- [ ] Salary disparity between my full-time clinical counterparts/Under-compensation
- [ ] Malpractice/lawsuit
- [ ] Discrimination/biases against your gender/ethnicity/sexual orientation
- [ ] Sexual harassment
- [ ] Balancing family and work responsibilities
- [ ] Balancing clinical, research, and educational responsibilities
- [ ] Satisfactory professional advancement
- [ ] Lack of support/resources by my chair/institution to apply for a career development award
- [ ] COVID-19 delaying/halting research projects
- [ ] Childcare
- [ ] Administrative burdens
- [ ] Other (please specify)

* 30. What are the TOP TWO most important factors to you in selecting a position/career?

- [ ] Opportunities to do research
- [ ] Opportunities for patient care
- [ ] Opportunities for BOTH research and patient care
- [ ] Opportunities to teach
- [ ] Opportunities for community service
- [ ] Opportunities for interactions with trainees
- [ ] Ability to balance work and personal life
- [ ] Financial security
- [ ] Autonomy
- [ ] Prestige
- [ ] Childcare resources
- [ ] Childcare flexibilities
- [ ] Other (please specify)

* 31. In which area do you intend to spend the majority of your professional time? Please check the MOST likely/Highest priority one.

- ○ Education
- ○ Basic research
- ○ Clinical research
- ○ Translational research
- ○ Clinical duties
- ○ Therapeutics/diagnostics development
- ○ Advocacy
- ○ Administration
- ○ Other (please specify)

[ ]

* 32. In the last six months, have you considered leaving academic medicine within the next 2 years?

- ○ Yes
- ○ No
- ○ If considering leaving, please describe your top reasons for this:

[ ]

* 33. In the next 5 years, how likely do you think it is that you will stay in academic medicine?

- ○ 100%
- ○ >75%
- ○ 50-75%
- ○ 25-50%
- ○ <25%
- ○ Other (please specify):

[ ]

* 34. Thinking about the reasons that might lead you to consider leaving your position, check all that apply

- ☐ I am unhappy, stressed, or otherwise less than satisfied with my current position.
- ☐ I am drawn to, excited by, or otherwise attracted to a different position.
- ☐ Burnout
- ☐ Funding challenges
- ☐ Undercompensation
- ☐ I would not consider leaving.
- ☐ Other (please specify):

[ ]

35. Would joining an organization dedicated to both advocacy for funding, policy changes and provides career development content/networking for early-career physician-scientists be of interest to you?

- ◯ Yes
- ◯ No
- ◯ Not sure

36. Thank you for your time in helping to advocate for physician-scientists. Please let us know if you have any feedback, comments or suggestions

[ ]
