# Supplementary material for: Evaluating factors impacting early career physician-scientists’ decisions to continue research careers in the United States of America
Source: BMC Med Educ. 2025 Apr 17;25:564. doi: 10.1186/s12909-025-07144-4 (PMC12007356; doi:10.1186/s12909-025-07144-4)
Supplement: Supplementary file 2 — Supplementary Material 2 [file 12909_2025_7144_MOESM2_ESM.docx]

**Supplemental Table 1** Respondents Distribution Across Different Specialties

| **Specialty** | | **Respondents N = 230 (%)** |
| --- | --- | --- |
| Primary Care/Medicine-Based Subspecialties | Family Medicine | 1 (0.4%) |
|  | Internal Medicine | 9 (4%) |
|  | Cardiology | 18 (8%) |
|  | Gastroenterology | 13 (6%) |
|  | Geriatric | 2 (1%) |
|  | Infectious disease | 15 (7%) |
|  | Rheumatology | 11 (4%) |
|  | Pulmonology/Critical Care | 22 (10%) |
|  | Hematology/Oncology | 30 (13%) |
|  | Allergy/immunology | 1 (0.4%) |
|  | Endocrinology | 8 (3%) |
|  | Pediatrics | 16 (7%) |
|  | Medicine-Pediatrics | 1 (0.4%) |
|  | Palliative Care | 1 (0.4%) |
| Surgical Subspecialties | Plastic surgery | 1 (0.4%) |
|  | Trauma surgery | 2 (1%) |
|  | Ophthalmology | 2 (1%) |
|  | Urology | 2 (1%) |
|  | Ob/Gyn | 1 (0.4%) |
|  | General Surgery | 4 (2%) |
|  | Transplant surgery | 2 (1%) |
|  | Plastic surgery | 1 (0.4%) |
|  | Trauma surgery | 2 (1%) |
| Diagnostics | Medical Genetics | 4 (2%) |
|  | Pathology | 16 (7%) |
|  | Dermatology | 3 (1%) |
|  | Radiology | 9 (4%) |
|  | Radiation Oncology | 2 (0.4%) |
|  | Psychiatry | 5 (2%) |
|  | Neurology | 20 (8%) |
|  | Child Neurology | 2 (1%) |
| Acute Care Subspecialties | Anesthesiology | 2 (1%) |
|  | Emergency Medicine | 2 (1%) |

Table 1

This table summarizes the distribution of respondents across various specialties with a detailed breakdown within each group
