# Supplementary material for: Evaluating factors impacting early career physician-scientists’ decisions to continue research careers in the United States of America
Source: BMC Med Educ. 2025 Apr 17;25:564. doi: 10.1186/s12909-025-07144-4 (PMC12007356; doi:10.1186/s12909-025-07144-4)
Supplement: Supplementary file 3 — Supplementary Material 3 [file 12909_2025_7144_MOESM3_ESM.docx]

**Table 2** States Distribution Across Different Geographic Regions

| **Geographic Regions** | **States** |
| --- | --- |
| Midwest | Illinois, Iowa, Nebraska, Ohio, Michigan, Kansas, Missouri, Minnesota, Nort Dakota, South Dakota |
| Northeast | Connecticut, DC, Delaware, Maine, Maryland, Massachusetts, New Hampshire, New Jersey, New York, Pennsylvania, Rhode Island, Vermont |
| Northwest | Idaho, Montana, Oregon, Washington, Wyoming |
| South | Oklahoma, Texas |
| Southeast | Alabama, Arkansas, Florida, Georgia, Kentucky, Louisiana, Mississippi, North Carolina, South Carolina, Tennessee, Virginian, West Virginia |
| Southwest | Arizona, California, Colorado, Nevada, New Mexico, Utah |
| Other | No reported specific location |
