# Supplementary material for: Evaluating factors impacting early career physician-scientists’ decisions to continue research careers in the United States of America
Source: BMC Med Educ. 2025 Apr 17;25:564. doi: 10.1186/s12909-025-07144-4 (PMC12007356; doi:10.1186/s12909-025-07144-4)
Supplement: Supplementary file 4 — Supplementary Material 4 [file 12909_2025_7144_MOESM4_ESM.docx]

**Supplemental table 3** Distribution of Respondents Considering Leaving their Research Careers

| **Specialty** | | **Respondents N = 114** **(%)** |
| --- | --- | --- |
| Primary Care/Medicine-Based Subspecialties | Allergy/immunology | 1 (1%) |
|  | Cardiology | 8 (7%) |
|  | Endocrinology | 3 (3%) |
|  | Family Medicine | 1 (1%) |
|  | Gastroenterology | 4 (4%) |
|  | Geriatric | 2 (2%) |
|  | Hematology/Oncology | 15 (13%) |
|  | Infectious disease | 12 (11%) |
|  | Internal Medicine | 2 (2%) |
|  | Medicine-Pediatrics | 2 (1%) |
|  | Palliative Care | 1 (1%) |
|  | Pediatrics | 7 (5%) |
|  | Pulmonology/Critical Care | 13 (11%) |
|  | Rheumatology | 6 (6%) |
| Surgical Subspecialties | General Surgery | 1 (1%) |
|  | Ophthalmology | 2 (2%) |
|  | Plastic surgery | 1 (1%) |
|  | Transplant and HPB Surgery | 1 (1%) |
|  | Trauma surgery | 1 (1%) |
| Diagnostics | Child Neurology | 1 (1%) |
|  | Dermatology | 1 (1%) |
|  | Medical Genetics | 3 (3%) |
|  | Neurology | 8 (7%) |
|  | Pathology | 10 (9%) |
|  | Psychiatry | 2 (2%) |
|  | Radiation Oncology | 1 (1%) |
|  | Radiology | 4 (4%) |
| Acute Care Subspecialties | Anesthesiology | 1 (1%) |

This table summarizes the distribution of respondents considering leaving their research careers across various specialties with a detailed breakdown of specialties within each subgroup.
