# Supplementary material for: Evaluating factors impacting early career physician-scientists’ decisions to continue research careers in the United States of America
Source: BMC Med Educ. 2025 Apr 17;25:564. doi: 10.1186/s12909-025-07144-4 (PMC12007356; doi:10.1186/s12909-025-07144-4)
Supplement: Supplementary file 5 — Supplementary Material 5 [file 12909_2025_7144_MOESM5_ESM.docx]

**Supplemental table 2** Graduation Year from Clinical Training Prior to Seeking Full-time Position

| **Year of response** | **Number of respondents (N=185)** | **Percentages (%)** |
| --- | --- | --- |
| Before 2020 | 82 (44%) | 44% |
| 2020 or later | 103 (56%) | 56% |
